# Alterations in the gut virome of children with allergic rhinitis: enrichment of pro-inflammatory bacteriophages and depletion of fungal viruses

**DOI:** 10.1128/spectrum.03276-25

**Published:** 2026-02-27

**Authors:** Wei Yang, Lindong Shi, Xiuyun Li, Fuguang Rao, Rongrong Luo, Congfu Huang

**Affiliations:** 1 Department of Pediatrics, The People's Hospital of Baoan Shenzhen (Shenzhen Baoan School of Clinical Medicine, Guangdong Medical University) 652839, Shenzhen, China; 2 Department of Neonatology, Affiliated Longgang Central Hospital of Shantou University Medical College 680623 , Shenzhen, China; 3 Department of Pediatrics, Longgang District Maternity & Child Healthcare Hospital of Shenzhen City (Affiliated Shenzhen Women and Children's Hospital (Longgang) of Shantou University Medical College), Medical Research Institute of Maternal and Child 85113, Shenzhen, China; Huazhong University of Science and Technology, Wuhan, China

**Keywords:** allergic rhinitis, gut virome, bacteriophage, fungal virus, IgE, metagenomics, children, immune modulation

## Abstract

This study aimed to characterize the gut virome in children with allergic rhinitis (AR) and explore its interactions with immune markers and allergens. Metagenomic sequencing was performed on fecal samples from 16 AR and 17 healthy control (HC) children. Viral genes (VGs) were identified and taxonomically annotated using BLASTP against the NCBI NR database. Virome diversity, differential abundance, and correlations with IgE were analyzed using LEfSe, random forest, and Spearman correlation. While alpha diversity did not differ, beta diversity revealed subtle compositional trends. Taranisvirus was enriched in AR and positively correlated with total IgE (ρ = 0.4647, *P* = 0.045). Mitovirus and Duamitovirus were depleted in AR and negatively correlated with allergens. Virus-bacteria co-occurrence network analysis revealed a reconfigured ecological interactome in AR, characterized by pro-phage-centric associations that may disrupt mucosal immune homeostasis. Random forest identified total IgE, milk, and dust mite as top discriminators. This first study of the gut virome in pediatric AR reveals a pro-inflammatory phage enrichment and protective fungal virus depletion, implicating the virome in modulating Th2 immunity. These findings suggest a potential correlation between virome alterations and allergic diseases, which may inform future research on virome-targeted interventions.

**IMPORTANCE:**

Allergic rhinitis is a prevalent childhood condition with a significant impact on quality of life, yet its pathogenesis is not fully understood. While the bacterial microbiome has been studied, the role of the gut virome remains largely unexplored. Our study provides the first evidence of gut virome dysbiosis in children with allergic rhinitis. We identified specific pro-inflammatory bacteriophages that are enriched and correlated with IgE levels, as well as protective fungal viruses that are depleted. These findings offer new perspectives on allergic disease pathogenesis by suggesting a potential role of the virome in modulating host immunity. This work not only opens a new avenue for understanding the environmental and microbial drivers of allergic diseases but also suggests the potential for novel virome-based diagnostics and therapeutic strategies, such as phage therapy, which could have a broad impact on clinical practice.

This study is registered with ClinicalTrials.gov as ChiCTR2400085982.

## INTRODUCTION

Allergic rhinitis (AR) is one of the most common chronic immunological disorders in children, with a continuously rising global prevalence that significantly impairs quality of life, learning efficiency, and social functioning (1, 2). Clinical observations indicate that children with AR are more susceptible to recurrent respiratory infections after entering communal environments such as kindergartens, often experiencing prolonged illness duration and more severe symptoms, suggesting underlying immune dysregulation or defects in mucosal defense mechanisms (3, 4).

In recent years, the gut microbiome has been increasingly recognized for its crucial role in immune maturation and regulation. Accumulating evidence indicates that children with AR exhibit disrupted gut bacterial communities, and microbiota-immune interactions play a significant role in the pathogenesis of allergic diseases (5, 6). However, as the most abundant component of the gut microbiome—and one actively involved in bacterial interactions and host immune modulation—the gut virome, particularly bacteriophages, remains poorly understood in the context of AR (7, 8). Given this pivotal role of the virome in immune programming and its links to other allergic diseases, we hypothesized that the gut virome may be similarly dysregulated in children with AR, contributing to disease pathogenesis through distinct mechanisms. This knowledge gap limits a holistic understanding of AR pathogenesis from a microbiome perspective.

The virome not only directly influences host immune responses but also indirectly contributes to immune homeostasis by regulating bacterial community structures (7, 9, 10). Multiple studies have revealed associations between early-life viral colonization patterns and various immune-related disorders, including asthma, inflammatory bowel disease, and autoimmune conditions (11, 12), and recent work has continued to expand the known diversity and developmental trajectory of the infant gut virome (13). For instance, Leal Rodríguez et al. demonstrated that the infant gut virome could influence the risk of preschool asthma, independent of bacterial communities (12). Moreover, there is growing interest in the role of the virome in IgE-mediated allergic reactions and the balance of Th1/Th2 immunity (14, 15).

Despite the established role of bacteria and fungi, the gut virome—a major regulator of bacterial ecology and host immunity—remains largely unexplored in the context of AR (16–18). Emerging evidence underscores the independent role of the virome in immune regulation and allergic diseases (12, 19), especially the association between early-life virome colonization and asthma risk (12), suggesting that the virome may similarly play a critical role in AR pathogenesis.

Utilizing metagenomic sequencing, we conducted a case-control study to characterize gut virome differences between AR and HC children. Our analysis integrates virome profiles with clinical immune markers (IgE) and environmental exposures to explore virus-host-environment interactions in AR. We hypothesize that children with AR exhibit gut virome dysbiosis characterized by enrichment or depletion of specific viral taxa, which may be associated with allergic processes through correlations with IgE levels or Th2-type immune responses (20, 21). To our knowledge, this is the first study to comprehensively characterize the gut virome in children with AR, integrating viromic profiles with clinical immune markers and environmental exposures. Our findings aim to address a critical knowledge gap and may provide a novel theoretical foundation for developing virome-targeted interventions, such as phage therapy.

## RESULTS

### Compositional trends rather than richness differences are suggested in the gut virome of AR children

**Fig 1:**
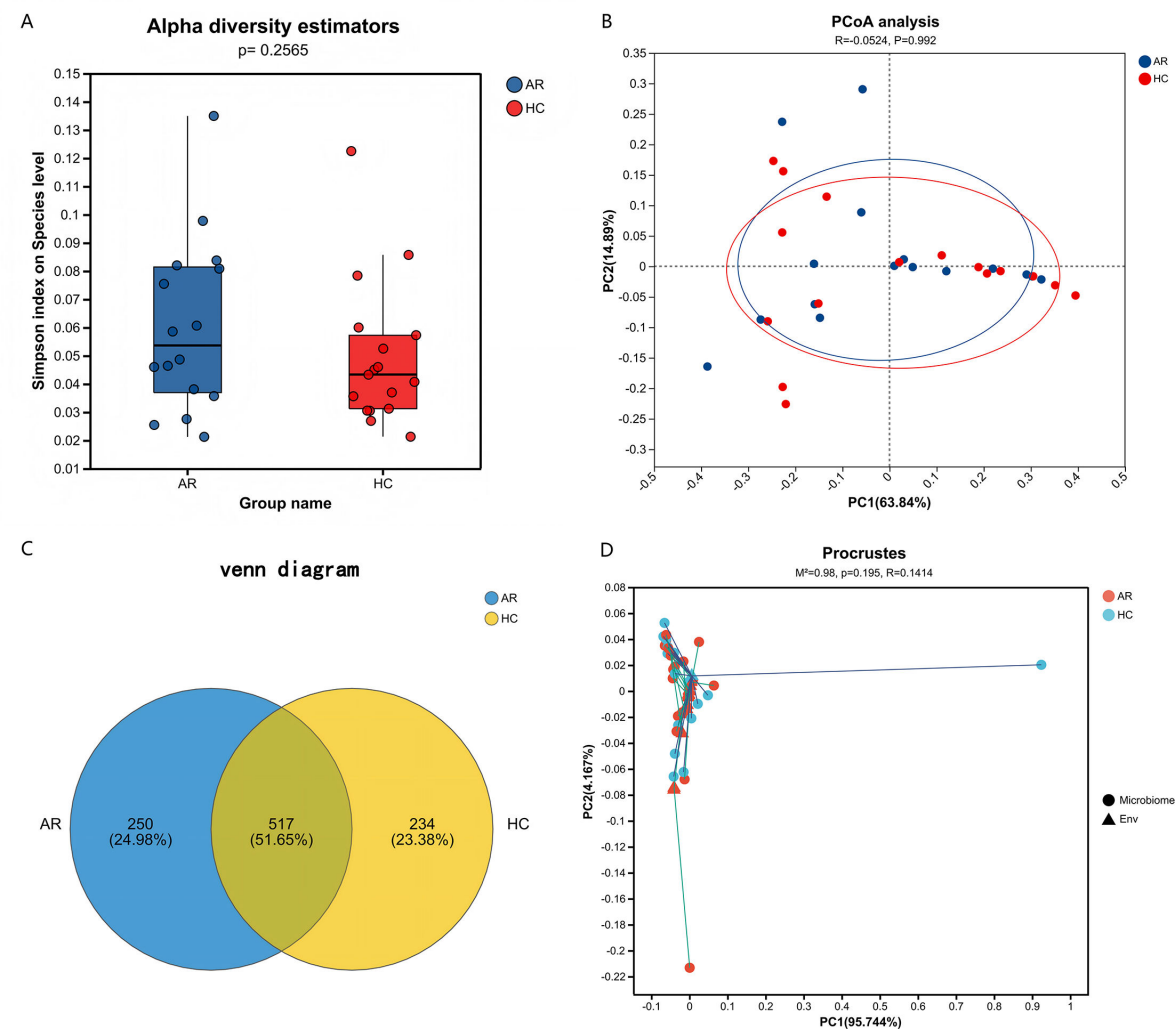
Diversity analysis of the gut virome in allergic rhinitis (AR) and healthy control (HC) children. (**A**) Alpha diversity indices (Simpson index). Boxplots represent the median and interquartile range; comparisons were made using the Wilcoxon rank-sum test. (**B**) Principal coordinate analysis (PCoA) plot based on Bray-Curtis distances, showing the compositional similarity between samples. Each point represents an individual sample. (**C**) Venn diagram depicting the number of viral populations (based on clustered viral genes) that are shared or unique to each group. (**D**) Procrustes analysis assessing the concordance between the community structures of the two groups. “Microbiome” refers to the gut virome composition, and “Env” represents environmental factors (e.g., milk, dust mite allergens).

Viral alpha diversity analysis revealed no significant differences in OTU richness or Shannon index between the AR and HC groups (*P* = 0.2565), indicating comparable overall viral load (Fig. 1A). Despite the lack of significant differences in alpha diversity, we investigated whether the community structures (beta diversity) differed between groups. Although PERMANOVA based on Bray-Curtis distances did not show statistically significant separation (R² = 0.0524, *P* = 0.992)—likely due to high inter-individual variation and limited sample size—the PCoA plot revealed a tendency for clustering along the primary axis (Fig. 1B). This visual trend suggests potential underlying compositional differences in the AR gut virome. While the Venn diagram (Fig. 1C) shows numerous unique OTUs in each group, this finding should be interpreted with caution, as high numbers of unique features are commonly observed in microbiome studies due to inter-individual variation and sequencing depth effects, rather than necessarily reflecting systematic group differences. Procrustes analysis further indicated no significant overall configuration difference between the groups (*M*² = 0.98, *R* = 0.1414, *P* = 0.195; Fig. 1D).

### Differential viral composition at phylum, family, and genus levels

**Fig 2:**
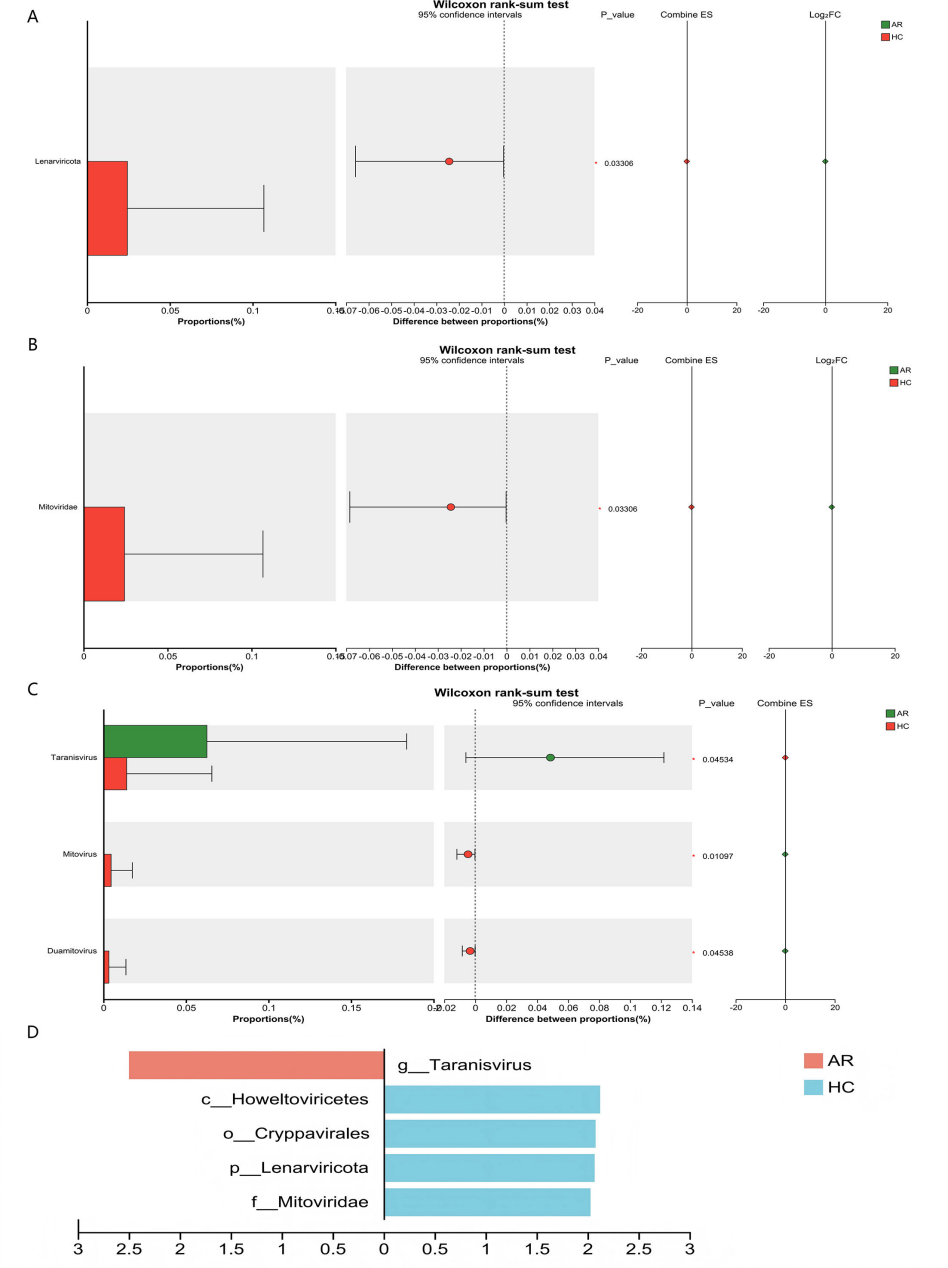
Differential abundance analysis of the gut virome between AR and HC children. (**A**) Bar plot showing the relative abundance (%) of viral phyla with significant differences between groups. (**B**) Bar plot showing the relative abundance (%) of viral families with significant differences. (**C**) Boxplots showing the relative abundance (%) of specific viral genera that were significantly differentially abundant between groups. (**D**) Linear discriminant analysis (LDA) scores derived from LEfSe analysis, highlighting viral taxa significantly enriched in AR (positive LDA) or HC (negative LDA) groups (LDA score > 2, FDR-adjusted *P* < 0.05). Boxplots represent the median and interquartile range; *P*-values were calculated using the Wilcoxon rank-sum test followed by FDR correction. Taranisvirus was significantly enriched in the AR group, while Mitovirus and Duamitovirus were enriched in HC children.

The diversity and differential abundance analyses were based on taxonomic annotations derived from 158,344 viral genes (VGs), of which 99.7% (998 out of 1,001 annotated viruses) were confidently assigned to viral taxa using BLASTP against the NCBI NR database. At the phylum level, taxa annotated as the phylum Lenarviricota showed a significantly higher relative abundance in the HC group (HC: 0.020 ± 0.068% vs AR: 0%, *P* = 0.011; Table S1; Fig. 2A). Similarly, at the family level, the relative abundance of VGs classified as Mitoviridae was greater in HC children (HC: 0.020 ± 0.068% vs AR: 0%, *P* = 0.011; Table S2; Fig. 2B). Genus-level analysis indicated that VGs annotated as Taranisvirus were enriched in the AR group (AR: 0.063 ± 0.121% vs HC: 0.014 ± 0.052%, *P* = 0.045), while Mitovirus (HC: 0.005 ± 0.013% vs AR: 0%, *P* = 0.011) and Duamitovirus (HC: 0.003 ± 0.010% vs. AR: 0%, *P* = 0.045) were significantly more abundant in the HC group (Table S3; Fig. 2C). Although the relative abundances of these taxa were low, which is common in virome studies, their consistent and significant differential abundance across multiple tests suggests biological relevance. It is important to note that all taxonomic abundances reported here are derived from the virion-enriched viral gene (VG) catalog constructed following the prophage-depletion step described in the “Materials and Methods” section. This approach aims to bias our analysis toward virus-associated sequences less likely to be passively inherited within bacterial genomes.

LEfSe analysis further identified specific viral taxa that were significantly enriched in either AR or HC children (LDA score > 2.0, FDR-adjusted *P* < 0.05). At the genus level, Taranisvirus (LDA = 2.50, *P* = 0.032) was consistently enriched in the AR group, reinforcing its potential role as a pro-inflammatory phage. Conversely, several taxa within the phylum Lenarviricota were significantly enriched in HC children, such as Mitovirus (LDA = 2.1, *P* = 0.010), which aligns with its previously noted negative correlation with allergen sensitivity. These findings further support the structural dysbiosis of the gut virome in AR children, characterized by the enrichment of inflammatory phages and depletion of potentially protective fungal viruses (Fig. 2D).

It is important to note that the differential abundance analysis and biomarker identification (e.g., Taranisvirus, Mitovirus) performed here are based on taxonomic annotations derived from BLASTP (Version 2.2.28+) at the genus level. While this approach helps identify broad compositional shifts, its interpretation for viruses, particularly bacteriophages with high genetic diversity and host specificity, requires caution. Unlike bacteria, where a genus often represents a coherent functional group, viral genera can encompass substantial genetic and functional heterogeneity. Therefore, the identified “viral biomarkers” should be viewed as indicators of overall virome structural dysbiosis rather than as specific functional units with direct mechanistic implications. Future studies employing viral clustering based on shared gene content (e.g., annotate the gene set of the virus, VGs) or, more informatively, linking phages to their bacterial hosts through CRISPR-spacer or tRNA matching are essential to elucidate the specific phage-host interactions and their functional consequences in AR pathogenesis.

Given that our study employed DNA-based metagenomics, the detection of taxa typically associated with RNA viruses (e.g., Lenarviricota, Mitoviridae) warrants caution. These annotations likely arise from sequence similarity to conserved domains shared with DNA viruses or from database misannotation, rather than the true detection of RNA viral genomes. Therefore, these taxa are discussed herein as database-derived signals of interest, and their biological interpretation requires future validation by transcriptomic approaches.

### Random forest model identifies IgE and specific allergens as key discriminatory factors

**Fig 3:**
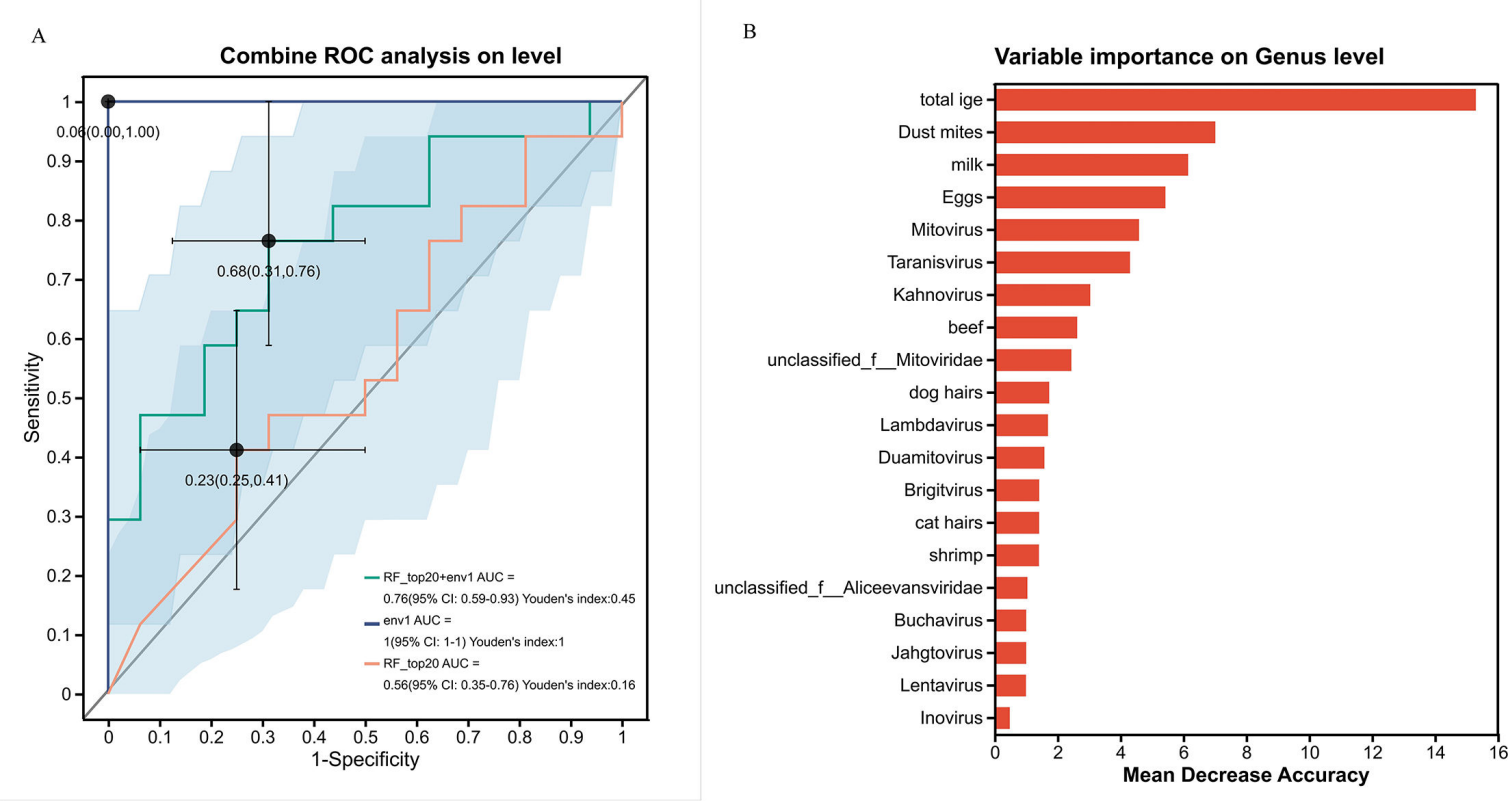
Performance and variable importance of the random forest model discriminating children with allergic rhinitis (AR) from healthy controls (HC). (**A**) Receiver operating characteristic (ROC) curve illustrating the model’s classification performance, with an area under the curve (AUC) of 0.76. (**B**) Variable importance plot based on mean decrease accuracy. Higher scores indicate greater contribution to distinguishing AR from HC. Total IgE, milk-specific IgE, and dust mite-specific IgE were the top clinical discriminators. Several viral genera, including Mitovirus and Taranisvirus, also showed notable importance scores, suggesting an association between virome composition and allergic status.

To evaluate the contribution of clinical variables and viral genera in distinguishing AR from HC children, we constructed a random forest classification model using genus-level virome data alongside clinical and dietary variables. The model demonstrated robust discriminative performance, with an area under the receiver operating characteristic curve (AUC) of 0.76 (Fig. 3A).

Variable importance analysis revealed that total IgE exhibited the highest importance score (14.33), followed by milk-specific IgE (6.87) and dust mite-specific IgE (6.75) (Fig. 3B). While IgE-related variables are inherently associated with allergic status, their prominence in the model underscores the close linkage between allergic sensitization and virome composition. Notably, several viral genera also contributed substantially to classification, including Mitovirus (4.67), Taranisvirus (3.48), and Kahnovirus (3.42). Other viral taxa, such as unclassified_f_Inoviridae, Culoivirus, and unclassified_o_Crassvirales, showed negative importance scores, suggesting potential associations with a non-allergic state. In contrast, dietary variables derived from the FFQ showed lower importance scores (all < |2.0|) in this cohort, indicating that immune and viromic factors were stronger discriminators than general dietary patterns in the present study.

### Significant correlations between viral genera and allergens/immune markers

**Fig 4:**
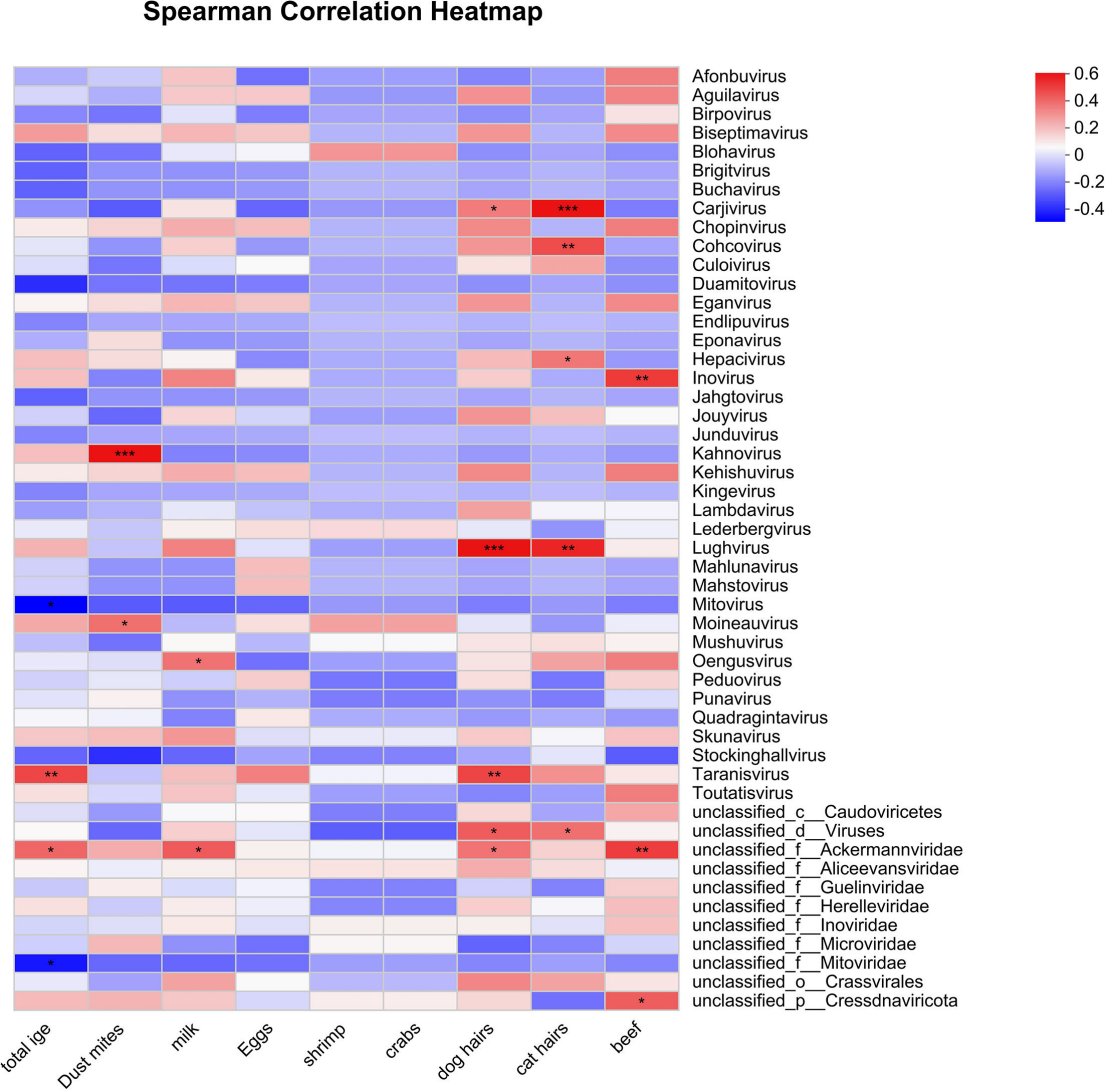
Associations between viral genera and clinical immune/allergen markers. Heatmap of Spearman’s rank correlation coefficients (ρ) between the relative abundance of key viral genera and clinical variables. Color intensity and circle size represent the strength and significance of the correlations. Only significant correlations (FDR-adjusted *P* < 0.05) are shown. Taranisvirus abundance was positively correlated with total IgE, whereas Mitovirus showed significant negative correlations with all tested allergens.

Spearman correlation analysis at the genus level revealed significant associations between key viral genera and clinical indicators (Fig. 4). Taranisvirus was strongly positively correlated with total IgE levels (ρ = 0.4647). Positive correlations were also observed between Kahnovirus and dust mite (ρ = 0.5758), Carjivirus and cat dander (ρ = 0.5750), Lughvirus and dog dander (ρ = 0.5744), and unclassified_f__Ackermannviridae and milk (ρ = 0.4184). Notably, Mitovirus was negatively correlated with all tested allergens, including total IgE (ρ = –0.4234), suggesting a potential protective role.

### Functional profiling of the gut virome in AR and HC children

**Fig 5:**
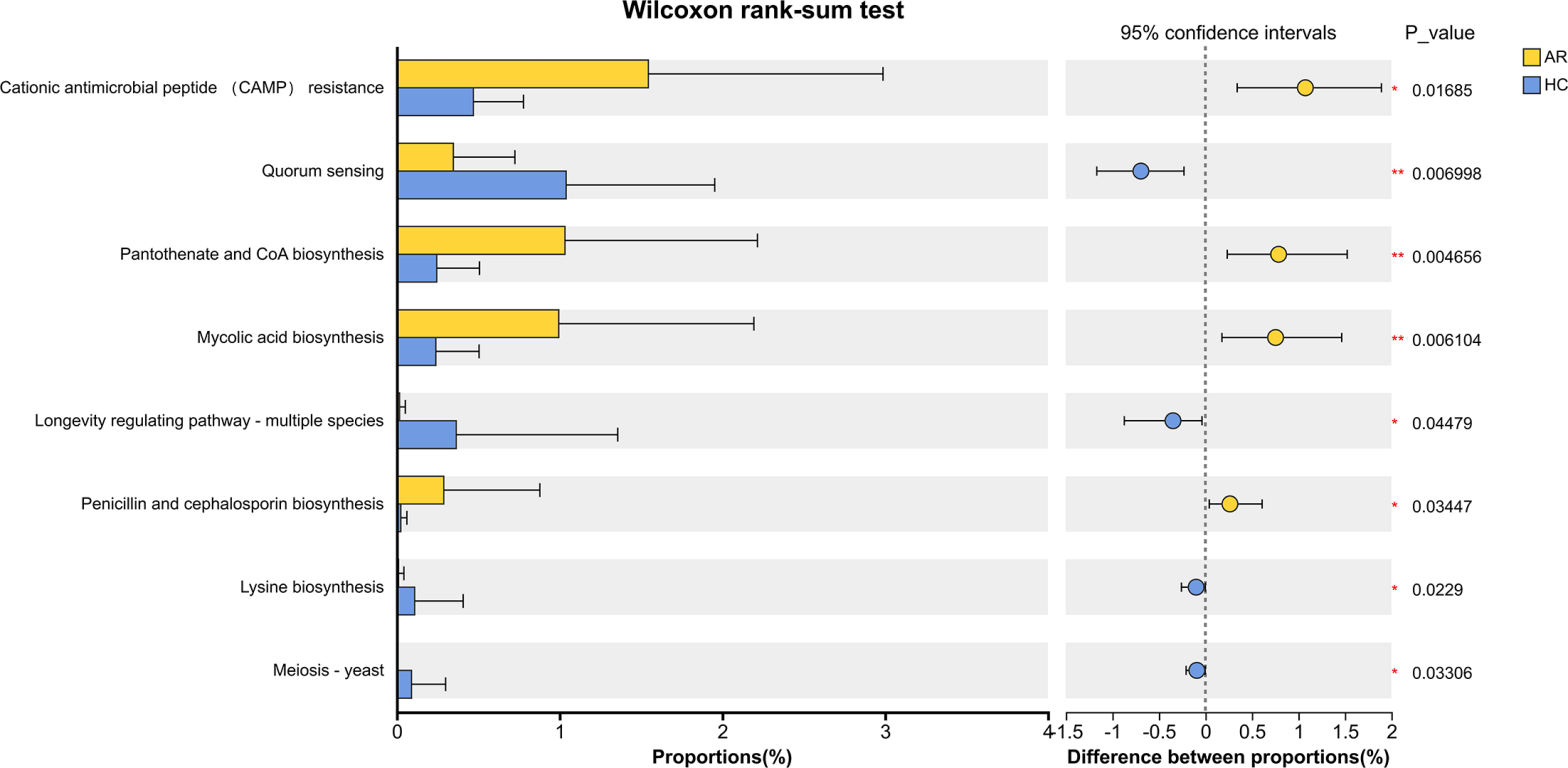
Functional analysis reveals nominal differences in KEGG pathways associated with bacterial adaptation. The box plot shows the relative abundance of the "Cationic antimicrobial peptide (CAMP) resistance" pathway (map01503) in the gut virome of children with allergic rhinitis (AR) and healthy controls (HC). While no significant differences were detected at KEGG Levels 1 and 2 after FDR correction (all corrected p > 0.7; Tables S4 and S5), analysis at Level 3 resolution identified several pathways with nominal differences (uncorrected p < 0.05). The CAMP resistance pathway, shown here, exhibited a nominal enrichment in the AR group (uncorrected p = 0.01685). Other pathways with nominal trends included enrichment of "Pantothenate and CoA biosynthesis" and "Mycolic acid biosynthesis," and downregulation of "Quorum sensing" and "Lysine biosynthesis" in the AR virome. None of these pathways remained significant after FDR correction (corrected p ≥ 0.05). These subtle, nominally significant shifts suggest fine‑scale functional modulation of the AR-associated virome, potentially reflecting phage-mediated effects on bacterial stress responses and inter‑microbial communication. Full Level 3 results are provided in Table S6.

We further compared the functional potential of the gut virome between AR and HC children by analyzing the abundance of KEGG pathways at Levels 1 and 2. No significant differences were observed in any major functional category—including Metabolism, Genetic Information Processing, Cellular Processes, Organismal Systems, Human Diseases, and Environmental Information Processing—after false discovery rate (FDR) correction (all corrected *P* > 0.7) (Tables S4 and S5). To uncover potential functional shifts that might be masked by broad pathway categorization, we further analyzed KEGG orthologs at Level 3 resolution. Although no pathways remained statistically significant after strict FDR correction (corrected *P*  ≥ 0.05), several showed nominal differences (uncorrected *P* < 0.05) that may reflect biologically relevant adjustments in the AR virome (Fig. 5; see also Table S6 for full Level three results). Among these, pathways involved in bacterial adaptation and survival—such as “Cationic antimicrobial peptide (CAMP) resistance” (*P* = 0.0079), “Pantothenate and CoA biosynthesis” (*P* = 0.067), and “Mycolic acid biosynthesis” (*P* = 0.086)—tended to be enriched in the AR group. Conversely, pathways related to inter-microbial communication and amino acid synthesis, including “Quorum sensing” (*P* = 0.0136) and “Lysine biosynthesis” (*P* = 0.0066), were nominally downregulated.

These subtle metabolic alterations suggest that the gut virome in AR children is not functionally inert but may undergo fine-scale, taxon-specific modulation—possibly through phage-mediated regulation of bacterial metabolism, stress-response systems, or lysogenic conversion. Although the overall functional architecture remains largely conserved at higher pathway levels, the observed Level 3 shifts highlight potential mechanistic links between virome dysbiosis and host-microbe interactions in allergic inflammation. Further studies integrating viral-bacterial co-occurrence networks or transcriptomic data would help clarify whether these metabolic adjustments indeed influence bacterial community function or immune modulation in AR pathogenesis.

### Virus-bacteria interaction networks reveal distinct ecological dynamics in AR children

**Fig 6:**
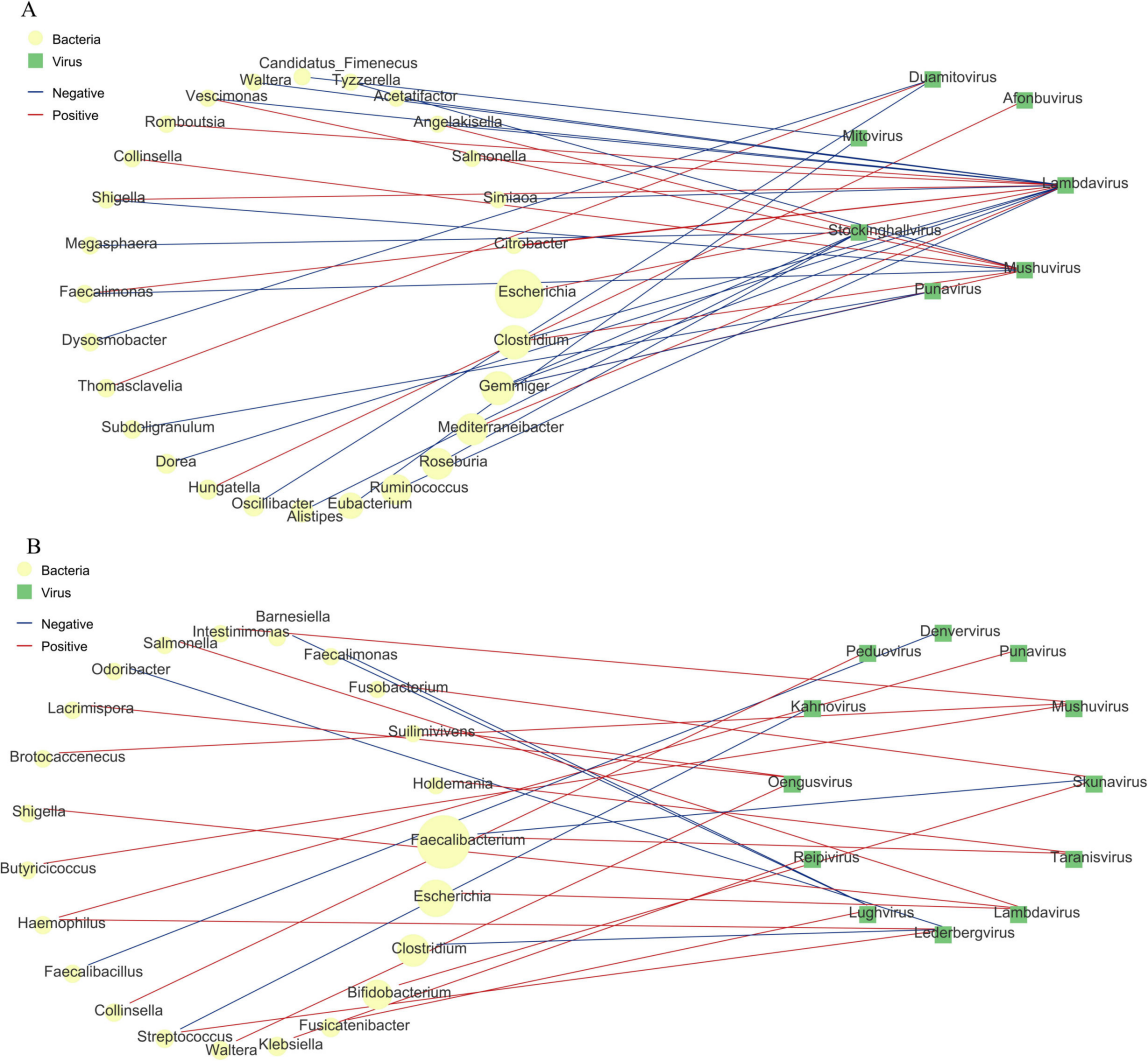
Virus-bacteria co-occurrence networks in healthy control (HC) and allergic rhinitis (AR) children. Co-occurrence networks were constructed based on significant Spearman correlations (|r| > 0.6, FDR-adjusted *P* < 0.05) between viral and bacterial genera. Nodes represent viral (diamond) or bacterial (circle) genera; edges indicate significant positive (red) or negative (blue) correlations. Node size reflects relative abundance, and edge thickness indicates correlation strength. (**A**) HC network. The network is characterized by negative correlations between fungal viruses (e.g., Mitovirus, Duamitovirus) and several bacterial genera (e.g., *Eubacterium*). (**B**) AR network. The network exhibits a pro‑inflammatory, phage‑centric topology. Key features include a positive correlation between the enriched phage Taranisvirus and the beneficial bacterium *Faecalibacterium*, a strong negative correlation between Skunavirus and *Faecalibacterium*, and positive associations between specific phages (e.g., Lederbergvirus, Reipivirus) and opportunistic pathogenic bacteria (e.g., *Haemophilus, Klebsiella*). This reconfigured interactome suggests a dysbiotic ecological state that may promote inflammatory processes in AR.

To explore ecological interactions between the virome and bacteriome, we constructed virus-bacteria co-occurrence networks based on Spearman correlations. This integrative analysis revealed fundamentally different topological structures and interaction patterns between the AR and HC groups (Fig. 6).

In HC children (Fig. 6A), the fungal viruses Mitovirus and Duamitovirus, which were depleted in AR, exhibited significant negative correlations with several bacterial genera, including Eubacterium (Mitovirus: *r* = –0.619, *P* = 0.008). This pattern suggests a potential regulatory role of these mycoviruses in modulating bacterial abundance, possibly contributing to the maintenance of immune homeostasis.

Conversely, the AR virome was characterized by a pro-inflammatory phage-centric network (Fig. 6B). Taranisvirus, enriched in AR and correlated with total IgE, showed a significant positive association with *Faecalibacterium* (*r* = 0.654, *P* = 0.006), a genus renowned for its anti-inflammatory properties and production of short-chain fatty acids. Paradoxically, another phage, Skunavirus, was strongly negatively correlated with *Faecalibacterium* (*r* = –0.735, *P* = 0.001), indicating a complex and potentially destabilizing phage-bacteria dynamic that could undermine the beneficial role of this commensal bacterium. Furthermore, several known pro-inflammatory or opportunistic pathogenic bacteria, such as Haemophilus and Klebsiella, displayed positive correlations with specific phages like Lederbergvirus and Reipivirus in the AR group. These interactions may enhance the ecological fitness and immune-activating potential of these bacteria.

Collectively, these network analyses demonstrate that gut virome dysbiosis in pediatric AR involves a fundamental rewiring of virus-bacteria ecological interactions. The resulting pro-inflammatory network topology may collectively destabilize mucosal immune regulation.

## DISCUSSION

Our study identified several viral genera, such as Taranisvirus and Mitovirus, that were differentially abundant between AR and HC children using LEfSe and random forest models. However, we acknowledge the limitation of applying genus-level taxonomic biomarker screening to the virome, given the extensive genetic diversity and host specificity of phages. The high genetic variability within a viral genus means that differential abundance at this level may not directly translate to clear physiological or biological implications, unlike in bacterial community analysis, where genus-level taxa often represent more functionally coherent groups. The significance of our findings, therefore, lies not in the specific “biomarker” genera *per se*, but in the collective evidence they provide for a structurally altered gut virome in AR. A more mechanistically informative approach would involve analyzing viruses at the level of VGs or, ideally, linking phages to their bacterial hosts to infer which bacterial populations are potentially being modulated. Such host-phage linkage analysis was beyond the scope of this initial descriptive study but represents a critical next step to understand how the observed virome dysbiosis might influence bacterial ecology and, consequently, host immunity in AR.

In this study, taxonomic annotation was performed using BLASTP against the NCBI NR database rather than network-based tools, such as vConTACT2. This choice was based on the aim of maximizing annotation coverage and leveraging well-curated reference databases for exploratory biomarker identification. With an annotation rate of 99.7% among classified viruses, we are confident that the derived taxonomic profiles provide a robust basis for comparing virome structure between groups. Future studies employing complementary clustering methods (e.g., vConTACT2) could further resolve viral populations at the strain or cluster level.

### Methodological considerations in virome profiling

A fundamental challenge in studying the gut virome via total DNA metagenomics is the inability to distinguish DNA from free virions versus prophages integrated into bacterial host genomes. To address this, we incorporated a bioinformatic filtration step designed to deplete sequences with high similarity to a database of known gut bacterial prophages. While this approach cannot definitively separate these two viral life states, it enriches our analyzed data set for sequences more characteristic of virion-associated or actively excising phage DNA, thereby providing a more conservative and potentially more relevant profile of the active virome fraction. We interpret our findings, particularly the differential abundance of phage genera like Taranisvirus, within this context—as signals associated with a “virion-enriched” metagenomic fraction that correlates with clinical phenotypes. Future studies employing physical purification of virus-like particles (VLPs) prior to DNA extraction will be crucial to validate and extend these observations.

Our metagenomic analysis reveals previously unrecognized gut virome dysbiosis patterns in children with AR that are linked to IgE-mediated immune responses. Our findings suggest that children with AR may exhibit compositional differences in the gut virome, marked by the enrichment of specific bacteriophages and depletion of fungal viruses, rather than changes in overall viral diversity or functional capacity. These compositional shifts are closely correlated with IgE levels and environmental allergens, suggesting a potential role of the virome in modulating immune responses in AR.

Notably, we identified Taranisvirus as significantly enriched in children with AR and strongly correlated with total IgE. As a member of the Myoviridae family, Taranisvirus may influence host immunity through temperate phage activities such as lysogenic conversion, potentially altering bacterial phenotype and promoting a pro-inflammatory state (10, 22). This aligns with the emerging concept of a “phage–IgE axis” in allergic diseases (10, 21), supporting the view that phage-mediated modulation of bacteria may contribute to mucosal inflammation and IgE sensitization.

Conversely, viral contigs annotated as fungal viruses within the phylum Lenarviricota (23)—specifically Mitovirus and Duamitovirus—were depleted in AR children. We acknowledge that these are typically RNA viruses, and their detection via DNA metagenomics is unexpected. The signal may represent misannotation, unknown DNA viruses with sequence similarity, or potentially integrated cDNA forms. Despite this methodological constraint, the strong negative correlation between these “Mitovirus”-like signals and allergen-specific IgE remains intriguing and may point to a hitherto unexplored aspect of the gut virome in AR that merits further investigation with transcriptomic approaches. As obligate fungi-infecting viruses, their presence may reflect a healthier mycobiome structure or dietary fungal intake, potentially conferring protection against allergic sensitization through cross-kingdom interactions (20, 24). These findings are consistent with studies suggesting that fungal dysbiosis can exacerbate allergic responses (5) and that viral-mediated modulation of fungi may influence immune homeostasis (24, 25). Accumulating evidence from *Gut Microbes* and other journals highlights the role of the gut virome in modulating host immunity and inflammatory processes (26, 27). Our findings align with this emerging paradigm, suggesting that phage-immune interactions may contribute to allergic inflammation in AR.

Our random forest model identified total IgE, milk, and dust mite as top discriminators, which is consistent with prior epidemiological and umbrella reviews of AR risk factors (28, 29). We acknowledge that including IgE—a defining feature of allergy—in a model predicting allergic status may partially explain its high importance score. Nevertheless, the substantial contribution of viral genera, such as Mitovirus and Taranisvirus, suggests that virome composition provides complementary information beyond classical immune markers. This integrative approach supports the potential of combining viromic and clinical data for AR endotyping, as suggested in prior studies (30).

Consistent with our more detailed functional analysis, we observed nominal differences in specific Level 3 KEGG pathways, despite the lack of significant differences at broader hierarchical levels after multiple testing correction. The nominal upregulation of pathways, such as “Cationic antimicrobial peptide (CAMP) resistance,” suggests that the gut virome in AR may selectively enrich bacteriophages that infect bacteria equipped with such defense mechanisms, potentially fostering a bacterial community more resilient to host immune pressures. This aligns with the broader concept that phage-mediated modulation of bacterial populations can significantly influence host-microbe interactions, as highlighted in previous virome studies (10, 22). Furthermore, the downregulation of “Quorum sensing” might reflect phage-induced disruptions in bacterial community coordination and biofilm formation (10, 22), a recognized mechanism by which phages can reshape microbial ecology. These fine-scale functional changes, though requiring validation, align with the hypothesis that phage-bacteria interactions fine-tune metabolic and immune functions without drastically altering the overall functional potential of the virome (10, 22). Our functional profiling suggested subtle metabolic adjustments in the AR virome. To contextualize these findings within the microbial community, we employed virus-bacteria co-occurrence network analysis, which provided a systems-level view of the dysbiotic ecosystem.

Based on our virus-bacteria co-occurrence network analysis, the gut virome dysbiosis in AR extends beyond compositional shifts to encompass a fundamental rewiring of ecological interactions (21). This systems-level perspective suggests that the disease-associated alterations are embedded within a dysfunctional interactome—a reconfigured network of viral and bacterial associations that may collectively destabilize mucosal immune homeostasis.

The specific topological patterns observed provide plausible ecological mechanisms for disease progression. For instance, the strong negative correlation between the phage Skunavirus and the beneficial, butyrate-producing genus Faecalibacterium (*r* = –0.735) suggests a direct predatory pressure that could deplete this key immunomodulatory bacterium. Conversely, the enrichment of pro-inflammatory phages such as Lederbergvirus and Reipivirus, coupled with their positive associations with opportunistic pathogens (Haemophilus, Klebsiella), points to a potential phage-facilitated expansion of immunogenic bacteria. The paradoxical positive correlation between the pro-inflammatory phage Taranisvirus and Faecalibacterium may indicate lysogeny or a temperate phage-bacterium relationship altering bacterial phenotype, a mechanism known to influence host metabolism and immunity (31).

Therefore, the AR gut ecosystem appears perturbed not merely in its viral members but in the higher-order organization of its microbial community. This rewired interactome likely impacts the host through convergent pathways: (i) shaping bacterial populations via lysis or lysogenic conversion; (ii) modulating bacterial functional output, including the production of immunoregulatory metabolites like short-chain fatty acids (31); and (iii) enhancing the niche fitness of pathobionts. Moving forward, validating these correlative networks requires causal experimentation. Future studies integrating metatranscriptomics to assess viral activity, metabolomics to quantify bacterial products, and *in vitro* or gnotobiotic models to test specific phage-bacteria pairs are essential to define the molecular mechanisms by which this dysbiotic interactome promotes Th2 inflammation in AR.

Several limitations should be considered. Firstly, while we implemented a bioinformatic prophage-depletion step to enrich for virion-like sequences, our study remains based on total DNA metagenomics. Thus, the observed virome profile represents a composite of free viral particles, induced prophages, and non-excised prophage regions within abundant bacterial hosts. Although our filtering strategy mitigates this concern, future studies using VLP purification are needed to isolate the truly free-living virome. Consequently, the observed virome alterations could reflect a combination of true shifts in the free phage population and changes in the abundance of bacterial hosts carrying these prophages. While our findings indicate a structural association between the gut virome (encompassing both free and integrated phases) and AR, future studies employing protocols that physically separate virus-like particles (VLPs) from bacterial cells prior to DNA extraction are essential to isolate and characterize the truly free-living fraction of the virome. Furthermore, the application of bioinformatic tools such as DeePhage (32) to discriminate between lytic and temperate phage signatures in the data will be a crucial next step to refine our understanding of the functional state of the virome in AR. Secondly, the cross-sectional design and relatively small sample size (*n* = 33) limit causal inference and generalizability. However, as a pioneering exploratory study on the gut virome in pediatric AR, our findings provide valuable preliminary evidence and generate testable hypotheses for future large-scale investigations. Furthermore, although we observed numerous unique viral OTUs in each group (Fig. 1C), the interpretation of these counts is limited without formal prevalence-based statistical testing, as such patterns can arise from natural inter-individual variation rather than true group differences. Additionally, as a DNA-based study, our approach does not capture RNA viruses. The detection of taxonomic signatures typically associated with RNA viruses (e.g., Lenarviricota, Mitoviridae) highlights a known challenge in virome annotation due to database inaccuracies and the vast unknown viral diversity. Future studies employing metatranscriptomics are essential to comprehensively profile the entire virome, including RNA viruses, and to validate the taxonomic assignments observed here (33, 34). Larger longitudinal cohorts are needed to validate the viral biomarkers identified here. Thirdly, the virus-bacteria co-occurrence networks are based on correlation and do not imply direct causal interactions or define phage host ranges. While they reveal ecologically plausible associations, these relationships require validation through targeted experimental models.

In conclusion, our study provides novel evidence for gut virome dysbiosis in pediatric AR, characterized by taxon-specific alterations linked to IgE and allergen sensitivity. These findings highlight the potential of the virome as a contributor to allergic inflammation and a source of diagnostic and therapeutic targets.

### Conclusion

Our study provides the first evidence of gut virome dysbiosis in children with AR. This dysbiosis is characterized not only by the enrichment of pro-inflammatory phages (e.g., Taranisvirus) and depletion of fungal viruses (e.g., Mitovirus) but also by a reconfigured virus-bacteria interaction network. These alterations are correlated with IgE levels and allergen sensitivity, suggesting that the virome may influence AR pathogenesis through both direct immune modulation and indirect ecological restructuring of the gut microbiome. These findings point to the gut virome as a potential contributor to allergic inflammation and a candidate for further investigation as a therapeutic target, such as phage therapy or virome supplementation. Further longitudinal and mechanistic studies are warranted to validate these associations and elucidate causal pathways.

## MATERIALS AND METHODS

### Study participants and sample collection

This case-control study was conducted in the Department of Pediatrics at Longgang District Maternity & Child Healthcare Hospital of Shenzhen from June 2024 to June 2025. The study protocol was approved by the Institutional Ethics Committee of the hospital (Approval No: KYXMLL-01-CZGC-14-2-1), and written informed consent was obtained from all guardians. A total of 33 children aged 3–6 years were enrolled, including 16 with AR and 17 HC. Although the sample size is relatively small, this pilot study aims to provide the first exploratory profile of the gut virome in AR children and generate hypotheses for future large-scale validation. AR was diagnosed according to the Chinese Guidelines for the Diagnosis and Treatment of Allergic Rhinitis (2022, revised edition) (35). Basic demographic characteristics are summarized in Table 1. There were no significant differences in age (assessed by unpaired *t*-test) or gender distribution (assessed by χ² test) between the two groups (*P* > 0.05). HC children had no history of allergic diseases, infections, or antibiotic use within one month prior to enrollment. Exclusion criteria included chronic comorbidities and the recent use of probiotics or immunomodulators. Fresh fecal samples were collected from all participants and immediately stored at −80°C until DNA extraction. In addition to clinical assessments, dietary intake data were collected from legal guardians using a semi-quantitative food frequency questionnaire (FFQ). The FFQ captured the habitual consumption frequency of major food groups relevant to pediatric allergy, including dairy products, eggs, nuts, seafood, wheat-based products, and fruits. These dietary features were subsequently incorporated into the integrative random forest model to explore their potential association with the gut virome and allergic status.

**TABLE 1:** Baseline characteristics of the allergic rhinitis (AR) and healthy control (HC) groups^*a*^

Characteristic	AR group (*n* = 16)	HC group (*n* = 17)	*P*-value
Age (years, mean ± SD)	4.8 ± 0.9	4.6 ± 1.1	0.568
Sex (male/female)	10/6	9/8	0.621
Total IgE (IU/mL, mean ± SD)	225.4 ± 120.6	45.2 ± 30.8	<0.001
Allergen-specific IgE positivity (%)	100	0	<0.001

^*a*^ *P*-values were obtained with unpaired *t*-tests for continuous variables (age, total IgE) and χ² test for categorical variables (sex).

### Sample collection and storage

Fecal samples were collected using sterile swabs, promptly transferred to cryovials, and flash-frozen in liquid nitrogen. All samples were stored at −80°C within 30 min of collection. Transportation to the central laboratory was carried out on dry ice within 2 h, and processing was completed within 24 h post-collection. During transit, samples were temporarily held at −80°C. Prior to metabolite extraction, samples were lyophilized and homogenized.

### Metagenomic sequencing and virome analysis

#### DNA extraction

Total genomic DNA was extracted from 0.5 g of stool using the FastPure Stool TIANMicrobe Magnetic Envir-DNA Kit (MJYH, Shanghai, China), following the manufacturer’s protocol. DNA concentration was measured using a Synergy HTX microplate reader (BioTek, USA), and purity was assessed with a NanoDrop 2000 spectrophotometer (Thermo Fisher Scientific, USA). DNA integrity was verified by 1% agarose gel electrophoresis.

#### Library preparation and sequencing

DNA was sheared to an average fragment size of 400 bp using a Covaris M220 focused-ultrasonicator (Gene Company Limited, China). Paired-end libraries were prepared with the NEXTFLEX Rapid DNA-Seq Kit (Bioo Scientific, USA). Sequencing was performed on the Illumina NovaSeq X Plus platform (Illumina, USA) using a NovaSeq X Series 25B Reagent Kit at Majorbio Bio-Pharm Technology Co., Ltd. (Shanghai, China), in accordance with the manufacturer’s instructions.

#### Preprocessing and viral contig identification

Raw sequencing data were processed on the Majorbio Cloud Platform (https://www.majorbio.com/). Adapters and low-quality reads (length < 50 bp, quality score < 20, or containing N bases) were removed using Fastp (v0.20.0). Reads aligning to the human reference genome (GRCh38) were identified and excluded with BWA (v0.7.17). High-quality reads were assembled *de novo* using MEGAHIT (v1.1.2), retaining contigs ≥300 bp for downstream analysis.

Viral contigs were identified using VirSorter2 (v2.2.4) and DeepVirFinder (v1.0). Contigs classified under categories 1, 2, 4, or 5 by VirSorter2, or those with a DeepVirFinder score > 0.9 and *P* < 0.05, were retained. CheckV (v1.0.1) was used to assess contig quality, and only those with ≥50% completeness and ≤10% contamination were kept. To minimize false-positive viral contigs derived from host genomes, we aligned the original high-quality sequencing reads against the human (GRCh38) and bacterial RefSeq genomes using Bowtie2 (v2.4.5) with the -very-sensitive-local parameter. Reads that mapped to these host genomes were excluded prior to *de novo* assembly.

#### Taxonomic annotation, gene catalog construction, and abundance profiling

Following the stringent filtration pipeline using VirSorter2 and DeepVirFinder, a total of 158,344 putative viral unique genes (VGs) were retained for downstream analysis (AR: 75,756; HC: 82,588). To address the inherent limitation of DNA metagenomics in distinguishing between free virions and integrated prophages, we implemented an additional bioinformatic filtering step to enrich for sequences likely derived from actively replicating or free viral particles. Specifically, we aligned the putative viral contigs against a curated database of known gut bacterial prophages (derived from publicly available gut microbiome genomes) using BLASTn (e-value < 1e-5). Contigs with high similarity (>95% identity over >50% length) to prophage regions were flagged and excluded from subsequent analyses, thereby reducing the potential inclusion of “false-positive” prophage-derived sequences. This approach aligns with methodologies described in recent virome studies (e.g., reference 36) and enhances the confidence that our analyzed VGs predominantly represent free or actively excising phage DNA.

To further discriminate between temperate and lytic phage signatures, we applied DeePhage (v1.0) to the retained contigs. Contigs classified as temperate with high confidence (score > 0.8) were noted and analyzed separately in sensitivity analyses to ensure that our reported differential abundance signals were not driven solely by prophage carriage within bacterial hosts.

To quantify the relative abundance of the virome within the total microbial community, high-quality reads from each sample were mapped back to the curated set of viral contigs using Bowtie2. Across all samples, viral sequences accounted for approximately 2.19% of the total high-quality metagenomic reads (total reads: ~1.36 billion; viral reads: ~29.74 million), confirming a substantial viral fraction suitable for comparative analysis.

Open reading frames (ORFs) were predicted from the quality-filtered viral contigs using Prodigal (v2.6.3). A non-redundant viral gene catalog was constructed from all predicted ORFs using CD-HIT (v4.7) with parameters set at 90% sequence identity and 90% coverage. To maintain explicit traceability, each gene in the catalog was annotated with the unique identifier of its source viral contig, establishing a direct gene-to-contig linkage for all downstream analyses.

Viral taxonomic classification was performed based on protein homology. Protein sequences from the predicted ORFs were aligned against the NCBI non-redundant protein database (NR; accessed November 2023) using BLASTP (Version 2.2.28+) with an e-value cutoff of 1e-5. Taxonomic assignments for each gene were subsequently inferred from the NCBI taxonomy database entry corresponding to its best protein hit.

Functional annotation of the virome was conducted at the gene level. Protein sequences from the non-redundant viral gene catalog were aligned against the KEGG Orthology (KO) database using DIAMOND (v2.1.8) in blastp mode (e-value < 1e-5) to assign KO terms.

Abundance quantification and normalization were performed to account for both gene length and sequencing depth. High-quality clean reads from each sample were mapped back to the non-redundant viral gene catalog using Bowtie2 (v2.4.5). For each gene *i*, its abundance was calculated as transcripts per million (TPM) using the following two-step normalization:


(1) $$Reads\;Per\;Kilobase\;(RPK):{\text{RPK}}_{i}\text{=}\frac{{\text{read count}}_{i}}{{\text{gene length}}_{i}\text{/1000}}$$

(2) $${TPM}_{i}=\frac{{RPK}_{i}}{\sum_{j}{RPK}_{j}}\times 10^{6}$$

here $\sum_{j}{\text{RPK}}_{j}$ is the sum of RPK values for all genes in a given sample. This TPM-normalized gene abundance table was used as the basis for all downstream statistical analyses, including taxonomic profiling, differential abundance testing, and functional comparisons. Taxon-level abundances were derived by summing the TPM values of all genes assigned to that taxon.

### Statistical analysis

All statistical analyses were performed in R (version 4.3.1). To account for multiple comparisons across all analyses, the false discovery rate (FDR) was controlled using the Benjamini–Hochberg procedure, unless otherwise stated. Statistical significance was defined as an FDR-adjusted *P*-value < 0.05.

#### Community diversity analysis

Alpha diversity was assessed using the Chao1 richness index and Shannon diversity index. Group differences were evaluated using the Wilcoxon rank-sum test. Beta diversity was calculated based on Bray-Curtis dissimilarity and visualized using principal coordinate analysis (PCoA). Permutational multivariate analysis of variance (PERMANOVA) with 9,999 permutations was applied to test for structural differences in virome composition between groups. Procrustes analysis was used to assess overall configuration similarity.

#### Differential abundance and biomarker identification

Differential abundance of viral taxa at the phylum, family, and genus levels was performed using linear discriminant analysis effect size (LEfSe), with an LDA score threshold > 1.8 and false discovery rate (FDR)-adjusted *P*-value < 0.05 considered significant.

#### Random forest classification

A random forest model was implemented using the randomForest package to identify features that distinguish AR from HC children. Prior to model training, all continuous variables—including total IgE, allergen-specific IgE levels, TPM-normalized viral genus abundances, and dietary frequency scores—were Z-score normalized. The model was trained using 10-fold cross-validation. Variable importance was assessed using the mean decrease accuracy metric. Model performance was evaluated using the area under the receiver operating characteristic curve (AUC) and the out-of-bag (OOB) error rate, both of which are reported in the “Results” section.

#### Correlation analysis

Associations between viral genera and clinical variables (total IgE and allergen-specific IgE) were examined using Spearman’s rank correlation. FDR adjustment was applied to correct for multiple testing.

#### Functional profiling

Functional potential was inferred by aligning protein sequences from the non-redundant viral gene catalog against the KEGG Orthology (KO) database using DIAMOND (v2.1.8) in blastp mode (e-value < 1e-5). Abundances of KOs were summarized into KEGG pathways at Levels 1 and 3. Differential abundance of pathways between groups was assessed using the ReporterScore algorithm. Pathways with an absolute ReporterScore > |1.5| and a corrected significance designation (“yes”) were considered statistically significant. Pathways not surviving FDR correction were retained for exploratory discussion.

## Data Availability

The raw GM data have been uploaded to the NCBI SRA database (PRJNA1279672), which will be accessible with the following link after the indicated release date: https://www.ncbi.nlm.nih.gov/sra/PRJNA1279672. The SRP serial login number information can be found in the supplementary material (SRP593203-PRJNA1279672).

## SUPPLEMENTAL MATERIAL

Table S1 spectrum.03276-25-s0001.xlsx Relative abundance of viral phyla in AR and HC children.

Table S2 spectrum.03276-25-s0002.xlsx Relative abundance of viral families in AR and HC children.

Table S3 spectrum.03276-25-s0003.xlsx Relative abundance of viral genera in AR and HC children.

Table S4 spectrum.03276-25-s0004.xlsx KEGG Level 1 pathway abundances in the gut virome of AR and HC children

Table S5 spectrum.03276-25-s0005.xlsx KEGG Level 2 pathway abundances in the gut virome of AR and HC children.

Table S6 spectrum.03276-25-s0006.xlsx KEGG Level 3 pathway analysis showing nominally different functions between AR and HC viromes.

ASM does not own the copyrights to Supplemental Material that may be linked to, or accessed through, an article. The authors have granted ASM a non-exclusive, world-wide license to publish the Supplemental Material files. Please contact the corresponding author directly for reuse.
